# Are Positive Mental Health and Social Support Protective Factors in the Development of Suicidal Ideation in Chronic Pain? A Longitudinal Study

**DOI:** 10.1111/sltb.70083

**Published:** 2026-03-03

**Authors:** Jan‐Luca Tegethoff, Beatrice Korwisi, Julia Kähler, Antonia Barke, Thomas Forkmann, Inken Höller

**Affiliations:** ^1^ Department of Clinical Psychology and Psychotherapy University of Duisburg‐Essen Essen Germany; ^2^ Department of Clinical Psychology and Psychotherapy Charlotte Fresenius Hochschule Düsseldorf Germany; ^3^ Department of Clinical Psychology and Psychological Intervention University of Duisburg‐Essen Essen Germany

**Keywords:** chronic pain, entrapment, positive mental health, social support, suicidal ideation

## Abstract

**Background:**

The “integrated motivational‐volitional (IMV) model of suicidal behavior” links the development of suicidal ideation (SI) to feelings of defeat and entrapment. Positive mental health and social support appear to be protective in this process. Both were tested as moderating factors between entrapment and SI in the at‐risk group of persons living with chronic pain (PLCP).

**Methods:**

*N* = 285 PLCP participated in an online survey, *n* = 161 again at a 4‐week follow‐up. Generalized linear models tested whether positive mental health and social support moderated associations between both internal and external entrapment and SI, cross‐sectionally. Further, both entrapment facets, positive mental health, social support and their interactions were tested as predictors of change for SI, longitudinally.

**Results:**

Cross‐sectionally, only positive mental health moderated the relationship between both entrapment facets and SI. Longitudinally, none of the factors or their interaction were able to predict change in SI over 4 weeks.

**Conclusion:**

This study confirmed entrapment as a proximal risk factor for SI in PLCP. It further supports positive mental health buffering the impact of entrapment on SI. However, these factors could not predict change in SI over 4 weeks. This study underlines a need for (micro)longitudinal studies on this topic.

## 1 Introduction

Suicidal ideation (SI) and suicidal behavior are global health concerns that often occur alongside various mental or physical health conditions. However, combinations of risk factors alone have shown limited success in predicting suicide (Franklin et al. 2017). The “integrated motivational‐volitional (IMV) model of suicidal behavior” by O'Connor (2011) and O'Connor and Kirtley (2018) assumes a complex interaction of central mediating and moderating factors in the development of SI and suicidal behavior. Its central pathway describes that (1) feeling defeated due to certain individual life circumstances may lead to (2) feelings of entrapment, which in turn can result in SI and ultimately in suicidal behavior. This path is supported by a considerable body of literature (e.g., Branley‐Bell et al. 2019; Oakey‐Frost et al. 2022; O'Connor et al. 2013; Tucker et al. 2016; Wetherall et al. 2019). The overall feeling of entrapment originally consisted of two aspects: external entrapment, which is the inability to see any possibility of escape from unbearable and uncontrollable external circumstances, and internal entrapment, which is the desire to escape one's own thoughts, feelings, or inner states, yet feeling unable to do so (Gilbert and Allan 1998). However, some studies have hypothesized that feelings of internal entrapment predominate in the development of SI. This suggests that these constructs should be differentiated in future research (De Beurs et al. 2019; Höller et al. 2021, 2022; Lucht et al. 2020; Owen et al. 2018; Wetherall et al. 2022).

The IMV model has already been studied in different subgroups, but one particularly vulnerable group has not yet been considered: persons living with chronic pain (PLCP) are at substantial risk of suicidal ideation and behavior (Kwon and Lee 2023; Tang and Crane 2006). Chronic pain describes persistent or recurring pain conditions over a period of 3 months and more (Treede et al. 2019). Examples of common chronic pain syndromes include chronic low back pain, migraine, neuropathic pain, or chronic visceral pain, such as endometriosis. In a recent meta‐analysis, Kwon and Lee (2023) reported a pooled two‐week prevalence of SI in PLCP of 25.87% (95%‐CI: 18.09%–34.50%), which is nearly seven times higher than the prevalence rates of the general European population (Castillejos et al. 2021). In the IMV model, pain‐related factors were initially assumed to play a role in the transition from SI to suicidal behavior as volitional moderators (O'Connor and Kirtley 2018). For example, a heightened pain tolerance may contribute to the acquired capability for suicide (Van Orden et al. 2010). However, chronic pain and its characteristics, such as pain intensity or pain‐related interference, are also part of the pre‐motivational condition that accounts for increased vulnerability of PLCP to develop SI as a consequence of defeat and entrapment (Bekrater‐Bodmann 2025; Kirtley et al. 2020). The current study takes into account the latter and aims to identify protective motivational moderators that reduce the risk of developing SI out of feelings of entrapment before suicidal behavior occurs. Studies examining protective characteristics regarding SI and suicidal behavior are scarce in PLCP and to the best of our knowledge no study investigated interactions between such characteristics and entrapment in this group. Furthermore, the mainly cross‐sectional nature of the existing studies is a well‐known limitation (Souza et al. 2024), yet there are very few longitudinal studies on protective factors. Some protective motivational moderators have already been identified in other populations, such as hope (Tucker et al. 2016), resilience (Li et al. 2020; Scowcroft et al. 2019; Wetherall et al. 2019; Zortea et al. 2020), positive self‐appraisals (Tegethoff et al. 2023), positive future thinking (Pollak et al. 2021; Rasmussen et al. 2010), sleep duration and quality (Littlewood et al. 2019), meaning in life or reasons for living (Moscardini et al. 2022). Enabling a person to adopt an alternative perspective (Teismann and Brailovskaia 2020) buffers the negative effect of entrapment on SI and may prevent SI initially and suicidal behavior subsequently (Johnson et al. 2011).

A protective factor that is already known in both clinical and non‐clinical samples appears to be positive mental health (Brailovskaia et al. 2019; Seet et al. 2024; Siegmann et al. 2019; Teismann et al. 2016; Teismann, Brailovskaia, et al. 2018; Teismann and Brailovskaia 2020). Positive mental health is defined as a higher‐level emotional, social and psychological well‐being (Lamers 2012). It combines two traditions of well‐being, implying that a person enjoys life and manages well to fulfill their needs in a hedonistic sense, while also feeling well‐equipped dealing with difficulties in life in a meaningful eudaimonic way (Deci and Ryan 2006; Keyes et al. 2002; Lukat et al. 2016). Positive mental health is more that just the absence of mental disorders or psychopathology (Keyes et al. 2002); it can even occur alongside mental health problems. In this way it may have a buffering effect, for example, on SI (Johnson et al. 2011). Such effects for positive mental health have been reported by Teismann and Brailovskaia (2020) in a non‐clinical sample in a cross‐sectional study.

According to the authors of the German Positive Mental Health Scale, the construct is accompanied by external factors, which in turn influence these inner experiences, for example, social support (Lukat et al. 2016). A recent meta‐analysis has clearly shown that social support is negatively associated with SI and suicidal behavior (Darvishi et al. 2024). Regarding PLCP, Shim et al. (2017) indicated a protective role of social support for SI in a sample of persons with rheumatic disease. Beyond PLCP, it has been found to buffer the effects of different risk factors on SI (López et al. 2022; Mackin et al. 2017; Oon‐arom et al. 2021; Siegmann et al. 2018), also regarding entrapment (Baker et al. 2024; Mclean 2020; Owen et al. 2022; Parra et al. 2021; Shelef et al. 2016). Rasmussen et al. (2010), who have already investigated social support moderating the relationship between entrapment and SI, were unable to find such moderation; they attributed this to the low prevalence and variance of social support in their sample. The present study may help to clarify the role of social support on the relationship between entrapment and SI in a specific risk group.

This study hypothesizes that (1a) internal entrapment is a significant cross‐sectional predictor of SI and that (1b) positive mental health and (1c) social support are significant cross‐sectional moderators of the relationship between internal entrapment and SI. Secondly, we hypothesize that (2a) external entrapment is a significant cross‐sectional predictor of SI and (2b) positive mental health and (2c) social support are cross‐sectional moderators of the relationship between external entrapment and SI. Additionally, we hypothesize that, even longitudinally, (3a) internal entrapment and (3b) the interaction of positive mental health and internal entrapment as well as (3c) the interaction of social support and internal entrapment predict change in SI over four weeks. Finally, we hypothesize that (4a) external entrapment and (4b) the interaction of positive mental health and external entrapment as well as (3c) the interaction of social support and external entrapment predict change in SI over 4 weeks.

## 2 Method

### 2.1 Participants

Participants were eligible if they lived with chronic pain (i.e., over at least 3 months; Treede et al. 2019), had sufficient language skills in German and were at least 18 years old. Participants were included on the basis of their self‐report that they had been suffering from pain for 3 months or more; they were not required to present a confirmed diagnosis of chronic pain. SI or suicidal behavior were assessed, but did not constitute an inclusion criterion. Participants, who did not answer the attention check item (“Enter 376 in the field below”) correctly, were excluded. *N* = 285 participants were included in the analyses. The baseline sample consisted of *n* = 252 (88.4%) females, *n* = 32 (11.2%) males, and one person who did not specify their sex (0.4%). The mean age of the participants was *M* = 41.2 (SD = 13.8) years at baseline. Most of the participants (*n =* 165; 57.9%) lived in a relationship, *n* = 116 (40.7%) participants were single and *n* = 4 (1.4%) were widowed. More than half the participants (*n* = 169; 59.3%) reported a current mental health problem, such as depression (*n* = 80; 28.1%), anxiety disorders (*n* = 23; 8.1%), or somatoform disorders (*n* = 19; 6.7%); the remaining participants reported that they had no mental health problems (*n* = 116; 40.7%). Of the participants, *n* = 79 (27.7%) specified that they were currently receiving psychotherapy, while *n* = 207 (72.6%) stated that they had received it in the past. In the longitudinal analyses, *n* = 161 persons were included, who fully participated in the follow‐up examination. At baseline, *n* = 185 (64.91%) reported an illness as the cause of their chronic pain, such as endometriosis (23.5%), spinal diseases (7.1%), for example, spinal disc herniations or scoliosis, arthritis (5.1%), fibromyalgia (4.6%), or migraine (3.5%). At baseline, pain durations ranged between 3 months and 55.8 years (*M* = 12.7 years; SD = 11.7). The majority of 77.19% received at least one treatment of their pain. Completer and non‐completer of the follow‐up survey did not significantly differ in the variables SI, external and internal entrapment, positive mental health and social support at baseline (*p >* 0.05). However, persons who completed the follow‐up were slightly younger (*M*_*C*_ *=* 39.49, SD *=* 13.12) than non‐completer (*M*_NC_ *=* 43.71, SD *=* 14.46), *t*(283) = 6.50, *p* = 0.01, and less burdened by the pain in case of pain intensity (*M*_*C*_ *=* 3.94, SD *=* 1.83; *M*_NC_ *=* 5.80, SD *=* 2.13; *t*(283) = 7.85, *p* < 0.001), pain‐related interference (*M*_*C*_ *=* 4.37, SD *=* 2.08; *M*_NC_ *=* 5.96, SD *=* 2.32; *t*(283) = 6.01, *p* < 0.001), and pain‐related distress (*M*_*C*_ *=* 4.21, SD *=* 2.33; *M*_*NC*_ *=* 6.04, SD *=* 2.36; *t*(283) = 6.44, *p* < 0.001) at baseline.

### 2.2 Procedure

The study was part of the project “A longitudinal study of chronic pain and its associations with suicidality and respective risk factors (ChronAS),” a collaboration between the University of Duisburg‐Essen and the Charlotte Fresenius Hochschule Düsseldorf. The participants were recruited through medical pain centers, patient organizations, and regional support groups across German‐speaking countries as well as via social networks (e.g., “Facebook”, “Instagram”) by posting flyers between March and June 2024. The online survey was released on the platform SoSci‐Survey (www.sosci‐survey.de). The full study consisted of a baseline assessment and one follow‐up assessment after 4 weeks. A preregistration including further information on the study (e.g., complete list of measures) is listed in the German Clinical Trials Register and the WHO International Clinical Trials Registry Platform (DRKS00033155). Prior to answering the questionnaire, all participants were informed about the study purpose, the voluntary nature of participating in the study, data storage, and data protection. Only participants who gave their informed consent, were able to continue to the survey. PLCP fully participating in both assessments received a voucher worth 15 € as an incentive. The study is in line with the Declaration of Helsinki and was approved by the Ethics Committee of the Institute of Psychology at the University of Duisburg‐Essen (EA‐PSY23/23/20112023).

### 2.3 Measures

#### 2.3.1 Entrapment Scale (ES‐d)

The German version of the entrapment scale (ES‐d; Trachsel et al. 2010) is a self‐report questionnaire. Originally developed by Gilbert and Allan (1998), 10 items assess external entrapment (e.g., “I can see no way out of my current situation”) and six items assess internal entrapment (e.g., “I feel trapped inside myself”) from 0 = “not at all” to 4 = “extremely” within the last 7 days. Thus, the external entrapment score (items 1–10) ranges from 0 to 40 and the internal entrapment score (items 11–16) from 0 to 24. Higher scores indicate more intense feelings of entrapment. Wichelhaus et al. (2025) confirmed good psychometric properties for the German version and good internal consistencies for the subscales internal (Cronbach's ⍺ = 0.85) and external entrapment (Cronbach's ⍺ = 0.84), being separate factors. In the present sample, the internal consistency for internal (Cronbach's ⍺ = 0.92; McDonalds ⍵ = 0.92) and external entrapment (Cronbach's ⍺ = 0.90; McDonalds ⍵ = 0.90) can be rated as excellent.

#### 2.3.2 Scale for Suicidal Ideation and Suicidal Behavior (SSEV)

The SSEV comprises a total of nine items, with six items assessing the frequency of different types of SI over the last 4 weeks (e.g., “During the past four weeks, I wished I was dead”) (Teismann et al. 2021). Items can be rated from 0 = “never” to 5 = “many times every day.” Additionally, one item asks about suicidal behavior in the past 4 weeks, one item assesses lifetime suicide attempts, while another item asks for the number of lifetime attempts. A score ≥ 1 of the first six items represented SI with total scores ranging from 0 to 30. Higher scores indicate more severe SI. The scale showed good internal consistency (Cronbach's ⍺ = 0.92; McDonald's ⍵ = 0.93), excellent test–retest reliability (*r* = 0.83) as well as robust construct validity in a mixed sample of German‐speaking individuals, including inpatients, outpatients, and online respondents (Teismann et al. 2021). The internal consistency of the six items, which assess SI over the previous 4 weeks, can be rated as good in the present sample (Cronbach's ⍺ = 0.86; McDonald's ⍵ = 0.87).

#### 2.3.3 Positive Mental Health Scale (PMH Scale)

The PMH Scale aims to assess the inner psychological and emotional factors of positive mental health in addition to contributing outer factors, such as social support (Lukat et al. 2016), and consists of nine items (e.g., “All in all, I am satisfied with my life.”). Each of the statements can be rated on a Likert scale from 1 = “not true” to 4 = “true,” with higher scores indicating higher levels of positive mental health. Lukat et al. (2016) reported a high mean internal consistency over different non‐clinical and clinical samples (Cronbach's α = 0.93). Due to good test–retest reliabilities across different samples (*r* = 0.74–0.81), equivalence tests, and the results of a sensitivity analysis, the authors evaluate the scale to be invariant across samples and over time, but sensitive to detect changes in clinical samples. The scale showed a good construct validity. The internal consistency in the present sample was excellent (Cronbach's ⍺ = 0.92; McDonald's ⍵ = 0.92).

#### 2.3.4 Oslo Social Support Scale—3 (OSSS‐3)

The OSSS‐3 in its German version is a three‐item assessment of social support (Dalgard et al. 1995; Kocalevent et al. 2018). The participants answer the first item (“How many people are so close to you that you can count on them if you have great personal problems?”) on a Likert scale from 1 = “none” to 4 = “5+,” the second item (“How much interest and concern do people show in what you do?”) from 1 = “none” too 5 = “a lot,” and the third item (“How easy is it to get practical help from neighbors if you should need it?”) from 1 = “very difficult” to 5 = “very easy.” Sum scores range from 3 to 14, with higher scores indicating more social support (Kocalevent et al. 2018). Kocalevent et al. (2018) pointed to a robust construct validity. The authors reported an acceptable internal consistency (Cronbach's ⍺ = 0.64) with regard to the brevity of the scale. The internal consistency in the present sample can also be rated as acceptable (Cronbach's ⍺ = 0.60; McDonald's ⍵ = 0.61).

### 2.4 Pain Characteristics

Pain‐related factors were assessed by using a section of the pain history taken from the German Pain Questionnaire (Petzke et al. 2020). Moreover, using 11‐point rating scales, participants were asked to rate their pain‐related interference (“How much has your pain impaired you in the last week (on average)?”), pain‐related distress (“How severe was your emotional distress due to your pain in the last week (on average)?”) as well as pain intensity (“How severe was your chronic pain in the last week (on average)?”) during the last 7 days from 0 = no interference/distress/pain to 10 = extreme interference/distress/pain (Treede et al. 2019).

### 2.5 Statistical Analyses

Statistical analyses were conducted with R (R Core Team 2021) and R Studio version 2024.09.1 (RStudio Team 2023), using the packages *haven* (Wickham et al. 2015), *lavaan* (Rosseel et al. 2012), *semTools* (Jorgensen et al. 2012), *interactions* (Long 2019), *correlation* (Makowski et al. 2020) and *lm.beta* (Behrendt 2014). The package *effectsize* (Ben‐Shachar et al. 2020) was used to estimate the semi‐partial eta squared as an effect size. An a priori power analysis using G*Power in version 3.1.9.7 (Faul et al. 2009; Mayr et al. 2007) indicated that a minimum sample size of *N* = 123 participants was required to test the most complex longitudinal linear regression models, considering the given number of predictors and covariates, assuming a moderate effect size (cf. Teismann and Brailovskaia 2020) and adjusting for multiple testing (1‐β = 0.80, α_adj._ = 0.0125, effect size *f* ^2^ = 0.15). Scale scores, means, standard deviations, and ranges for demographic variables were calculated, as well as bivariate correlations. Testing hypothesis (1a–c) and (2a–c), two moderated linear regression models were estimated with cross‐sectional data. Both models estimated whether positive mental health and social support significantly interact with (1a–c) internal entrapment respectively (2a–c) external entrapment and thus moderated the relationship between both entrapment facets and SI at baseline. To test hypotheses (3a–c) and (4a–c) and the predictive value of the protective factors for SI over 4 weeks, two generalized linear regression models were estimated, separately for (3a–c) internal entrapment and (4a–c) external entrapment. Positive mental health and social support as well as interaction terms of each factor with internal entrapment respectively external entrapment were considered while controlling for SI at baseline. All regression models were performed under control of pain duration, pain intensity, pain‐related interference, and pain‐related distress, which were treated as pre‐motivational factors, as well as age and sex. To receive a robust estimation of standard errors and confidence intervals a bootstrapping procedure (boot = 5000, seed = 654,321) has been performed using the PROCESS macro version 4.3.1 for R (Hayes 2023). Due to multiple testing, *p*‐values were adjusted using Bonferroni‐Holm method (Holm 1979).

## 3 Results

### 3.1 Descriptive Statistics and Correlations

Lifetime SI was reported by *n* = 187 (*n =* 65.6%) participants, while even *n* = 136 (47.7%) participants had SI within the last 4 weeks. Lifetime suicidal behavior was reported by *n* = 64 (22.5%) participants, while *n* = 5 (1.8%) showed recent suicidal behavior within the last 4 weeks. The descriptives of entrapment, recent SI, positive mental health, and social support are shown in Table 1. Further, Table 2 shows the correlations after Bonferroni‐Holm correction (Holm 1979). Recent SI was moderately to highly associated with internal (*r =* 0.49, *p <* 0.001) and moderately associated with external entrapment (*r =* 0.40, *p <* 0.001). Further, recent SI correlated moderately negatively with positive mental health (*r* = −0.42, *p* < 0.001) and slightly negatively with social support (*r* = −0.25, *p* < 0.001).

**TABLE 1:** Means, standard deviations, and ranges of the pain characteristics and main variables.

	Baseline (T1)				Four‐week follow‐up (T2)			
	(*N* = 285)				(*n* = 161)			
	*M*	SD	Min.	Max.	*M*	SD	Min.	Max.
Recent suicidal ideation	2.19	3.89	0	24	1.63	2.91	0	18
Entrapment	25.16	15.37	0	64	26.88	14.10	0	60
External entrapment	15.96	9.41	0	40	17.26	8.52	0	37
Internal entrapment	9.20	7.14	0	24	9.62	6.72	0	24
Positive mental health	13.23	5.69	0	27	—	—	—	—
Social support	8.94	2.16	4	14	—	—	—	—

*Note:* Recent suicidal ideation = Item score 1–6 of the Suicide Ideation and Behavior Scale, Entrapment = Score of the German Entrapment Scale (ES‐D), external entrapment = Item score 1–10 of the ES‐D, internal entrapment = Item score 11–16 of the ES‐D, Positive mental health = Positive Mental Health Scale score, Social support = Oslo Social Support Scale‐3 score.

**TABLE 2:** Correlations (Pearson's r) of defeat, entrapment, pain catastrophizing, and suicidal ideation at baseline.

	1	2	3	4	5	6	7	8	9
1. Entrapment	—								
2. External entrapment	0.95***	—							
3. Internal entrapment	0.91***	0.72***	—						
4. Suicidal ideation	0.47***	0.40***	0.49***	—					
5. Positive mental health	−0.57***	−0.53***	−0.53***	−0.42***	—				
6. Social support	−0.37***	−0.40***	−0.26***	−0.25***	0.47***	—			
7. Pain intensity	0.25***	0.25***	0.21**	0.22**	−0.19*	−0.16*	—		
8. Pain‐related interference	0.23**	0.22**	0.20**	0.24***	−0.24***	−0.16	0.79***	—	
9. Pain‐related distress	0.42***	0.39***	0.40***	0.32***	−0.42***	−0.24***	0.65***	0.64***	—
10. Pain duration	0.17*	0.21**	0.08	0.09	−0.05	−0.08	0.16*	0.18*	0.07

*Note:* *N* = 285 participants. Entrapment = Score of the German Entrapment Scale (ES‐D), external entrapment = Item score 1–10 of the ES‐D, internal entrapment = Item score 11–16 of the ES‐D, Recent suicidal ideation = Item score 1–6 of the Suicide Ideation and Behavior Scale, Positive mental health = Positive Mental Health Scale score, Social support = Oslo Social Support Scale‐3 score.

* *p* < 0.05, adjusted after Bonferroni‐Holm (1979).

** *p* < 0.01.

*** *p* < 0.001.

### 3.2 Cross‐Sectional Regression Analyses

**FIGURE 1:**
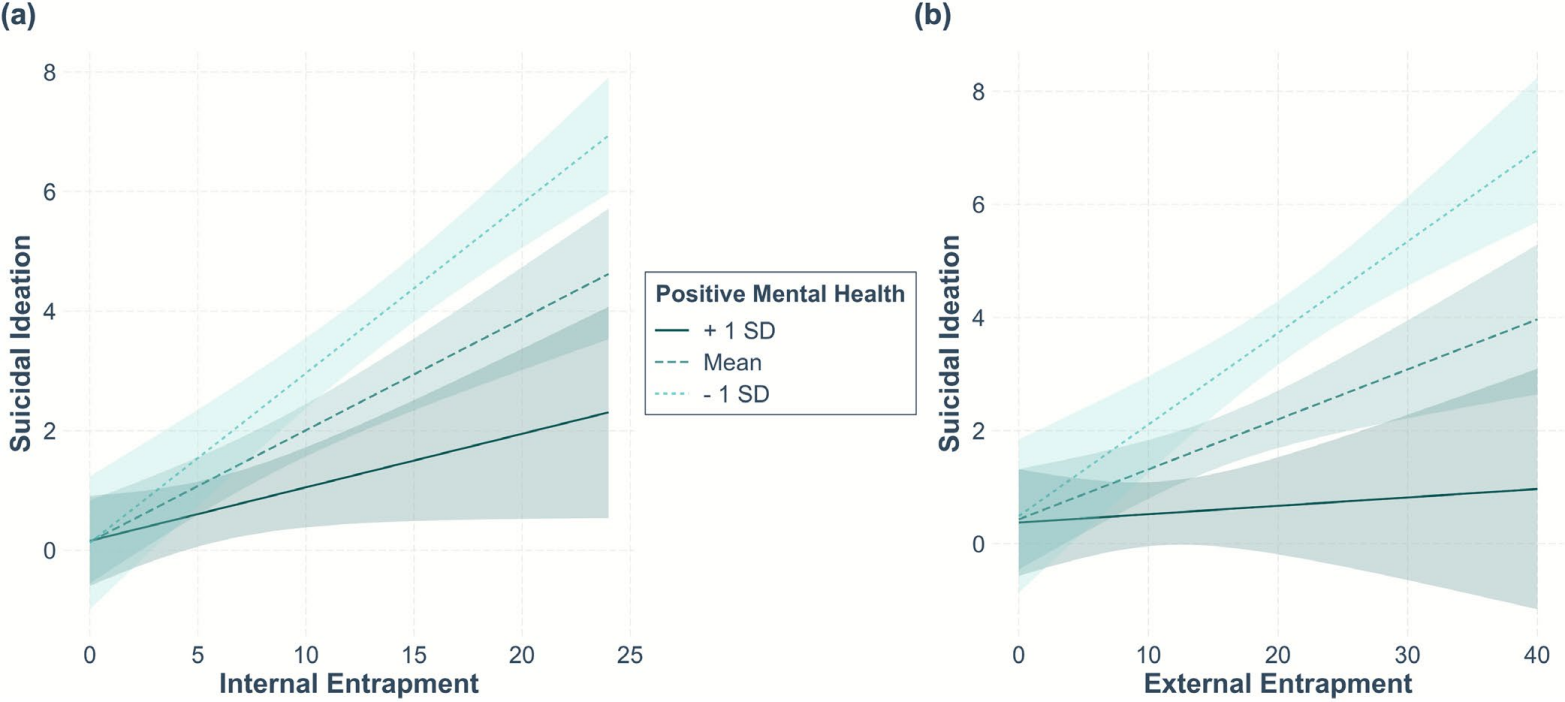
Cross‐sectional interactions of feelings of entrapment and positive mental health are presented affecting recent suicidal ideation (SI) at baseline. (a) There was a moderating impact of positive mental health reducing the effect of internal entrapment on SI when feelings of internal entrapment were high. Further, (b) the impact of external entrapment on SI was moderated, with high levels of positive mental health buffering the effect of increased feelings of external entrapment.

Table 3 presents the cross‐sectional models. Model 1 showed a suitable model fit, *F*(13,266) = 10.39, *p* < 0.001, *R* ^*2*^ = 0.34, 95% CI [0.25, 0.43], and explained 34% of variance in SI at baseline (Cohen 1988). Controlling for all the pain‐related factors, age, and sex, (1a) internal entrapment was a significant predictor of SI (β = 0.32, *p*_adj_ < 0.001, *sr* ^*2*^ = 0.25) with the largest contribution to the model, accounting for 25% of the variance. In addition to a significant negative main effect of positive mental health (β = −0.17, *p*_adj_ = 0.045, *sr* ^*2*^ = 0.02), (1b) positive mental health and internal entrapment showed a significant interaction effect on SI (β = −0.18, *p*_adj_ = 0.006, *sr* ^*2*^ = 0.02). Due to the moderation of positive mental health, hypotheses 1a and 1b could be confirmed. However, social support did not show a significant main effect on SI (β = −0.07, *p*_adj_ = 0.642, *sr* ^*2*^ = 0.01), nor (1c) a significant interaction with internal entrapment on SI (β = −0.03, *p*_adj_ > 0.999, *sr* ^*2*^ = 0.01). Thus, hypothesis 1c must be rejected. Model 2, testing the impact of external entrapment and its interaction with positive mental health and social support on SI, also showed a significant model fit, *F*(13,266) = 7.81, *p* < 0.001, *R* ^*2*^ = 0.28, 95% CI [0.19, 0.37]. A moderate proportion of variance of 28% was explained in the reported SI at baseline (Cohen 1988). External entrapment (2a) was a significant predictor of SI in the present sample (β = 0.17, *p*_adj_ < 0.001, *sr* ^*2*^ = 0.16) with the largest contribution to the model, accounting for 16% of the variance. Positive mental health showed a significant main effect (β = −0.25, *p*_adj_ = 0.001, *sr* ^*2*^ = 0.05) as well as (2b) a significant interaction effect with external entrapment on SI (β = −0.20, *p*_adj_ = 0.004, *sr* ^*2*^ = 0.02). Hypotheses 2a and 2b were therefore confirmed. Positive mental health moderated the relationship between external entrapment and SI. Figure 1 illustrates the moderating effects of positive mental health on the relationship of internal entrapment respectively external entrapment on the one side and SI on the other. Especially at higher levels of entrapment, high levels of positive mental health retrospectively buffered SI in the cross‐section.

**TABLE 3:** Parameter estimates for the cross‐sectional moderated linear regression models predicting suicidal ideation at baseline.

		Coefficients								Fit
		Unstandardized			Standardized	*t*	*p*	*p*_adj_.	*sr* ^*2*^	
		Est.	95% CI (Est.)	SE	*β*					
Model 1										*F*(13, 266) = 10.39, *p* < 0.001, *R* ^*2*^ = 0.34, 95% CI [0.25, 0.43]
	Intercept	1.15	−0.89, 3.23	1.04		1.09	0.277			
	IE	0.18	0.10, 0.25	0.04	0.32***	5.00	< 0.001	< 0.001	0.25	
	PMH	−0.12	−0.23, −0.02	0.01	−0.17*	−2.44	0.015	0.045	0.02	
	Social support	−0.12	−0.32, 0.11	0.11	−0.07	−0.99	0.321	0.642	0.01	
	IE × PMH	−0.02	−0.04, −0.001	0.01	−0.18**	−3.03	0.003	0.006	0.02	
	IE × Social support	−0.01	−0.06, 0.04	0.02	−0.03	−0.35	0.725	> 0.999	0.01	
	PMH × Social support	< 0.01	−0.05, 0.05	0.02	0.01	0.19	0.851	> 0.999	< 0.01	
	IE × PMH × Social support	< 0.01	−0.01, 0.01	< 0.01	0.01	0.20	0.840	0.840	< 0.01	
	Age	−0.01	−0.04, 0.03	0.02	−0.02	−0.29	0.770	> 0.999	< 0.01	
	Sex	−0.17	−1.29, 0.84	0.55	−0.01	−0.27	0.785	> 0.999	< 0.01	
	Pain duration	0.01	−0.03, 0.05	0.02	0.01	0.21	0.835	> 0.999	< 0.01	
	Pain intensity	0.06	−0.23, 0.36	0.15	0.04	0.41	0.681	> 0.999	0.01	
	Pain‐related interference	0.12	−0.16, 0.40	0.14	0.08	0.88	0.381	0.762	< 0.01	
	Pain‐related distress	0.02	−0.21, 0.25	0.12	0.01	0.13	0.897	0.897	< 0.01	
Model 2										*F*(13, 266) = 7.81, *p* < 0.001, *R* ^*2*^ = 0.28, 95% CI [0.19, 0.37]
	Intercept	1.31	−1.08, 3.62	1.21		0.30	0.765			
	EE	0.07	0.02, 0.13	0.03	0.17*	2.38	0.018	0.036	0.16	
	PMH	−0.18	−0.32, −0.07	0.07	−0.25***	−3.62	< 0.001	0.001	0.05	
	Social support	0.02	−0.21 0.27	0.12	0.01	0.12	0.902	0.902	< 0.01	
	EE × PMH	−0.02	−0.03, −0.004	0.01	−0.20**	−3.10	0.002	0.004	0.02	
	EE × Social support	0.01	−0.02, 0.03	0.01	0.02	0.31	0.760	> 0.999	< 0.01	
	PMH × Social support	< 0.01	−0.05, 0.05	0.02	0.01	0.31	0.754	> 0.999	< 0.01	
	EE × PMH × Social support	< 0.01	−0.003, 0.01	< 0.01	< 0.01	1.20	0.233	0.466	< 0.01	
	Age	−0.01	−0.05, 0.02	0.02	−0.05	−0.78	0.437	> 0.999	< 0.01	
	Sex	−0.14	−1.29, 0.95	0.57	−0.01	−0.21	0.832	> 0.999	< 0.01	
	Pain duration	< −0.01	−0.05, 0.04	0.02	< −0.01	−0.08	0.936	> 0.999	< 0.01	
	Pain intensity	0.04	−0.26, 0.38	0.16	0.02	0.23	0.822	> 0.999	0.01	
	Pain‐related interference	0.10	−0.14, 0.35	0.12	0.07	0.74	0.462	0.762	< 0.01	
	Pain‐related distress	0.10	−0.14, 0.34	0.12	0.07	0.83	0.406	0.812	< 0.01	

*Note:* Bootstrap samples *n* = 5000, seed = 654,321.

Abbreviations: EE = external entrapment, IE = internal entrapment, *p*_adj_. = adjusted *p*‐value after Bonferroni‐Holm correction, PMH = positive mental health.

*** *p*_adj_. < 0.001.

** *p*_adj_. < 0.01.

* *p*_adj_. < 0.05.

However, social support neither showed a significant main effect on SI (β = 0.01, *p*_adj_ = 0.902, *sr* ^*2*^ < 0.01), nor (2c) a significant interaction with external entrapment on SI (β = 0.02, *p*_adj_ > 0.999, *sr* ^*2*^ < 0.01). Thus, hypothesis 2c must also be rejected.

### 3.3 Longitudinal Analyses of Potential Predictors of Change

The longitudinal models (Table 4), which tested potential predictors of change of SI, rejected hypotheses 3 and 4. Model 3 showed a significant model fit (*F*(14,145) = 13.88, *p* < 0.001, *R* ^*2*^ = 0.57, 95% CI [0.46, 0.67]), but neither (3a) internal entrapment (β = −0.07, *p*_adj_ = 0.333, *sr* ^*2*^ < 0.01), nor (3b) an interaction of positive mental health and internal entrapment (β = −0.08, *p*_adj_ = 0.254, *sr* ^*2*^ < 0.01), nor (3c) an interaction of social support and internal entrapment (β = 0.01, *p*_adj_ > 0.999, *sr* ^*2*^ < 0.01), were able to predict change in SI over 4 weeks. Model 4 also showed a significant model fit, *F*(14,145) = 13.88, *p* < 0.001, *R* ^*2*^ = 0.57, 95% CI [0.46, 0.67]. However, neither (4a) external entrapment (β = −0.14, *p*_adj_ = 0.058, *sr* ^*2*^ < 0.01), nor (4b) an interaction of positive mental health and external entrapment (β = −0.04, *p*_adj_ = 0.588, *sr* ^*2*^ < 0.01), nor (4c) an interaction of social support and external entrapment (β = 0.03, *p*_adj_ > 0.999, *sr* ^*2*^ < 0.01), were able to predict change in SI over 4 weeks. At 57%, both models 3 and 4 explained a large proportion of variance (Cohen 1988) due to the large effect of SI (*sr* ^*2*^ = 0.50), which highly predicts itself over time.

**TABLE 4:** Parameter estimates for the longitudinal linear regression models predicting suicidal ideation at follow‐up.

		Coefficients								Fit
		Unstandardized			Standardized	*t*	*p*	*p*_adj_.	sr^2^	
		Est.	95% CI (Est.)	SE	β					
Model 3										*F*(14, 145) = 13.88, *p* < 0.001, *R* ^*2*^ = 0.57, 95% CI [0.46, 0.67]
	Intercept	−0.75	−2.06, 0.46	0.64		0.03	0.980			
	IE (T1)	−0.03	−0.09, 0.03	0.03	−0.07	−0.97	0.333	0.333	< 0.01	
	PMH (T1)	0.06	−0.03, 0.15	0.04	0.10	1.29	0.201	0.401	< 0.01	
	Social support (T1)	−0.16	−0.35, 0.02	0.10	−0.11	−1.56	0.120	0.360	0.02	
	IE × PMH (T1)	−0.01	−0.02, 0.01	0.01	−0.08	−1.15	0.254	0.254	< 0.01	
	IE × Social support (T1)	< 0.01	−0.03, 0.03	0.02	0.01	0.15	0.882	> 0.999	< 0.01	
	PMH × Social support	< 0.01	−0.03, 0.04	0.02	0.02	0.27	0.788	> 0.999	< 0.01	
	IE × PMH × Social support (T1)	< 0.01	−0.004, 0.01	< 0.01	0.08	0.98	0.328	0.656	< 0.01	
	Age	−0.01	−0.04, 0.01	0.01	−0.06	−1.023	0.308	> 0.999	< 0.01	
	Sex	0.49	−0.08, 1.10	0.30	0.06	0.99	0.326	0.978	< 0.01	
	Pain duration (T2)	0.03	−0.003, 0.07	0.02	0.13*	2.12	0.036	0.036	0.02	
	Pain intensity (T2)	−0.05	−0.28, 0.19	0.12	−0.02	−0.25	0.802	> 0.999	< 0.01	
	Pain‐related interference (T2)	0.02	−0.33, 0.40	0.19	< 0.01	0.01	0.996	> 0.999	< 0.01	
	Pain‐related distress (T2)	0.21	−0.08, 0.50	0.15	0.17	1.90	0.060	0.080	0.01	
	Suicidal ideation (T1)	0.57	0.40, 0.74	0.09	0.63***	9.35	< 0.001	< 0.001	0.50	
Model 4										*F*(14, 145) = 13.95, *p* < 0.001, *R* ^*2*^ = 0.57, 95% CI [0.46, 0.67]
	Intercept	−1.05	−2.40, 0.19	0.66		−0.13	0.895			
	EE (T1)	−0.04	−0.12, 0.01	0.03	−0.14	−1.91	0.058	0.058	< 0.01	
	PMH (T1)	0.04	−0.04, 0.13	0.04	0.08	1.06	0.291	0.402	< 0.01	
	Social support (T1)	−0.18	−0.39, 0.01	0.10	−0.13	−1.88	0.062	0.248	0.02	
	EE × PMH (T1)	< −0.01	−0.01, 0.01	0.01	−0.04	−0.543	0.588	0.588	< 0.01	
	EE × Social support (T1)	< 0.01	−0.02, 0.03	0.01	0.03	0.38	0.707	> 0.999	< 0.01	
	PMH × Social support (T1)	0.01	−0.03, 0.05	0.02	0.04	0.59	0.555	> 0.999	< 0.01	
	EE × PMH × Social support (T1)	< 0.01	−0.002, 0.01	< 0.01	0.07	0.86	0.391	0.466	< 0.01	
	Age	−0.01	−0.04, 0.01	0.01	−0.06	−0.90	0.369	> 0.999	< 0.01	
	Sex	0.58	−0.03, 1.26	0.32	0.07	1.17	0.242	0.968	< 0.01	
	Pain duration (T2)	0.04	0.001, 0.08	0.02	0.15*	2.46	0.015	0.030	0.02	
	Pain intensity (T2)	−0.05	−0.27, 0.19	0.12	−0.02	−0.24	0.809	> 0.999	< 0.01	
	Pain‐related interference (T2)	−0.01	−0.34, 0.40	0.19	−0.01	−0.07	0.944	> 0.999	< 0.01	
	Pain‐related distress (T2)	0.22	−0.07, 0.50	0.15	0.19	2.08	0.040	0.080	0.01	
	Suicidal ideation (T1)	0.60	0.42, 0.78	0.09	0.66***	10.15	< 0.001	< 0.001	0.50	

*Note:* Bootstrap samples *n* = 5000, seed = 654,321. *p*_adj_ = adjusted *p*‐value after Bonferroni‐Holm correction.

Abbreviations: EE = external entrapment, IE = internal entrapment, PMH = positive mental health, T1 = baseline, T2 = follow‐up.

*** *p*_adj_. < 0.001.

* *p*_adj_. < 0.05.

## 4 Discussion

In the present study, for the first time, positive mental health and social support were examined simultaneously as protective motivational moderators in the IMV model (O'Connor and Kirtley 2018) in the at‐risk group of PLCP. Stronger feelings of internal entrapment and external entrapment were associated with more severe SI. Additionally, higher levels of positive mental health and social support were correlated with less SI. According to hypothesis 1a and 1b, positive mental health significantly moderated the relationship between internal entrapment and SI in this sample. Thus, especially in cases of stronger feelings of internal entrapment, those with higher levels of positive mental health reported less severe SI. Social support (1c) did not show this effect in this study. Hypotheses 2a and 2b have also been confirmed. At baseline, positive mental health significantly moderated the relationship between external entrapment and SI. In cases of stronger feelings of external entrapment, higher levels of positive mental health came along with relatively less severe SI. However, social support (2c) did not show this effect. Hypotheses 3a‐c and 4a‐c must be rejected. Longitudinally, none of the factors or their interaction were able to predict change in SI in this sample of PLCP over the four‐week period.

The cross‐sectional findings of the present study confirm the late motivational phase of the IMV model for PLCP. While the present study shows effects of both internal and external entrapment on SI in PLCP, the moderate effect for internal entrapment on SI has been demonstrated, supporting its role as a primary predictor of SI (De Beurs et al. 2019; Höller et al. 2021, 2022; Lucht et al. 2020; Owen et al. 2018; Wetherall et al. 2022). However, the small to moderate effect of external entrapment on SI in this sample must be considered. The distinction between the two facets may not be as important as it is in other populations, for example in psychiatric samples in which internal entrapment seems to be the predominant facet (e.g., Höller et al. 2021; Lucht et al. 2020; Owen et al. 2018). Baker et al. (2024) came to a similar conclusion regarding external entrapment in the military context, where it also appears to play a significant role. The causes underlying a substantial influence of external entrapment in PLCP need to be investigated in future studies. It can only be assumed that maybe PLCP look at their pain and its consequences as more external than internal aspects of their current situation, from which they cannot escape. However, feelings of entrapment, that to our knowledge have not yet been investigated within this vulnerable group (cf. Kirtley et al. 2020), should therefore be considered in risk assessments and in future studies investigating SI and suicidal behavior in PLCP.

The IMV model addressed the buffering effect of protective factors proposed by Johnson et al. (2011) by postulating further moderating factors. In the literature, positive mental health has already been declared as a protective factor on suicidal outcomes in general (Brailovskaia et al. 2019; Teismann et al. 2016; Teismann, Forkmann, et al. 2018) and as a buffer for the amplifying effect that various risk factors show on SI, such as depression (Siegmann et al. 2018; Teismann, Brailovskaia, et al. 2018), perceived burdensomeness (Siegmann et al. 2019), and even entrapment (Teismann and Brailovskaia 2020). While distinguishing between internal and external entrapment, positive mental health cross‐sectionally buffered both entrapment facets in a similar way. About the mechanism behind this only speculations can be offered at this point. Positive mental health may contribute to a person's reasons for living as opposed to reasons for dying (Teismann, Forkmann, et al. 2018). This in turn could lead to an increase of suicidal ambivalence (cf. Teismann et al. 2024; Teismann and Forkmann 2024). In the present longitudinal analyses, positive mental health as well as its interaction with entrapment could not predict change in SI over 4 weeks. This issue could be due to the assumed stability of the positive mental health construct (Lamers 2012). However, this does not mean that there can be no change in the individual psychological, emotional and social well‐being over time, due to, for example, life changes (Keyes et al. 2002; Lamers et al. 2015). But change in positive mental health probably manifests over longer time frames and may therefore be unlikely to be reflected in this study in the change of SI over 4 weeks. The stability of positive mental health in PLCP over time, its effect on the development of SI over different time intervals and its consequences for suicidal ambivalence need to be further investigated in future studies.

Interestingly, social support did not show any effects in these analyses. This is in line with Rasmussen et al. (2010), who found no significant moderation of social support with regard to entrapment affecting SI, while positive future thinking was a significant moderator. Other studies found protective effects of social support. Shelef et al. (2016), who did not consider positive mental health in their study, found that soldiers with high entrapment levels showed less SI when social support was reported as high. Scardera et al. (2020) also showed that persons with higher levels of social support reported less likely SI and prospectively showed less suicidal behavior. Baker et al. (2024) found a moderating effect of tangible social support (i.e., practical help) on the relationship between internal entrapment and SI in a military sample. Appraisal support (i.e., seeking advice), strengthened the associations between external as well as internal entrapment and SI. Consequently, the type of social support perceived may differ in its protective effect; a factor that would be worth considering in future studies. Another perspective was taken by Owen et al. (2022), who reported an indirect effect of social support on change in SI by directly predicting changes in defeat and entrapment. This may explain why social support was not identified as a direct predictor of SI in this study either. In addition to Owen et al. (2022), it should be noted that social support may not only have already contributed to a person's defeat and entrapment level, but also to their positive mental health beforehand, which cannot be determined by this study design. Another strand of literature investigated the relationship between depression and SI. Seet et al. (2024) found that while positive mental health partially mediated the association between depression and SI, no effect of social support was found. Siegmann et al. (2018) also considered positive mental health next to social support and found mixed findings regarding social support by comparing a Chinese and a German student sample. While positive mental health was a moderator in both samples, social support was a significant moderator only in the Chinese sample. There also appear to be cultural differences in the protective role of social support against SI, which may also have influenced the results of this study.

This study is not without limitations. First, it is based on an online survey with retrospective self‐reports. Furthermore, the current sample was not strongly affected by SI on average, which resulted in a skewed distribution and limited interpretability. Additionally, the questionnaire on social support used in the present study with only three items may not be sufficient for this purpose. The quantity of supportive relationships in the immediate environment, which the questionnaire mainly asks about, is not necessarily an indicator of the different perceived qualities of the feeling of being socially supported in the current situation. Moreover, due to a rather small sample, only one aspect of the complex IMV model could be tested in the analyses. Finally, this longitudinal study only had one follow‐up assessment 4 weeks after baseline. On a positive note, this study considered that entrapment is increasingly regarded as two‐dimensional. Furthermore, recent SI was assessed by using a validated questionnaire assessing different stages of SI and not with a single item, as in some previous studies (Brailovskaia et al. 2019; Siegmann et al. 2018, 2019; Teismann, Brailovskaia, et al. 2018; Teismann, Forkmann, et al. 2018). Moreover, this study investigated an at‐risk sample consisting of PLCP, on which studies about SI, suicidal behavior, and respective protective and proximal risk factors, are scarce (Kirtley et al. 2020). However, it is well‐known that the development of SI and suicidal behavior as well as suicidal risk factors underlie short‐term fluctuations, even within days (Ben‐Zeev et al. 2012; Forkmann et al. 2018; Hallensleben et al. 2019; Kleiman et al. 2017; Kleiman and Nock 2018; Stenzel et al. 2020; van Ballegooijen et al. 2022). The manifestations of these also appear to vary between different risk groups (Kraiss et al. 2024). Future studies should assess interactions of risk and protective factors on SI in this group using micro‐longitudinal study designs. An ecological momentary assessment could be a suitable methodological approach to map the courses and interactions within suicidal development in real‐time. By using a questionnaire that distinguishes between the characteristics of perceived social support and by statistically testing indirect effects on SI via defeat, entrapment and positive mental health, interactions could finally be clarified.

## 5 Conclusion

Positive mental health may play a decisive protective role regarding the development of SI and suicidal behavior in PLCP. The present study cross‐sectionally supports the motivational phase of the IMV model (O'Connor and Kirtley 2018) and demonstrates a central role of entrapment as a proximal risk factor in this particular group. The role of social support in this process remains open. Micro‐longitudinal studies could provide new insights in the development of suicidal ideation and behavior in PLCP by identifying possible short‐term interactions of suicidal risk factors and those protective factors.

## Author Contributions

B.K. and I.H.: conceptualization. J.‐L.T., A.B., T.F.: contribution to the study design. B.K., I.H., J.‐L.T., J.K.: investigation. J.‐L.T., I.H.: methodology. J.‐L.T., I.H.: formal analysis. J.‐L.T.: writing original draft. B.K., I.H., J.K., T.F., A.B.: review and editing. I.H.: supervision. All authors have read and agreed to the version of this manuscript.

## Funding

We acknowledge support by the Open Access Publication Fund of the University of Duisburg‐Essen (project DEAL).

## Ethics Statement

The study is in line with the Declaration of Helsinki and was approved by the Ethics Committee of the Institute of Psychology at the University of Duisburg‐Essen (EA‐PSY23/23/20112023).

## Consent

Consent to participate in the study was given by actively clicking a checkbox, thereby confirming that the participant was of legal age and that the participant information above had been read and understood by the participant.

## Conflicts of Interest

The authors declare no conflicts of interest.

## Data Availability

The data that support the findings of this study are available on request from the corresponding author.
